# Regular recreational physical activity and risk of head and neck cancer

**DOI:** 10.1186/s12885-017-3223-7

**Published:** 2017-04-21

**Authors:** Chen-Lin Lin, Wei-Ting Lee, Chun-Yen Ou, Jenn-Ren Hsiao, Cheng-Chih Huang, Jehn-Shyun Huang, Tung-Yiu Wong, Ken-Chung Chen, Sen-Tien Tsai, Sheen-Yie Fang, Tze-Ta Huang, Jiunn-Liang Wu, Yuan-Hua Wu, Wei-Ting Hsueh, Chia-Jui Yen, Yu-Hsuan Lai, Hsiao-Chen Liao, Shang-Yin Wu, Ming-Wei Yang, Forn-Chia Lin, Jang-Yang Chang, Yi-Hui Wang, Ya-Ling Weng, Han-Chien Yang, Yu-Shan Chen, Jeffrey S. Chang

**Affiliations:** 1 Department of Nursing, National Cheng Kung University Hospital, College of Medicine, National Cheng Kung University, Tainan, Taiwan; 2 Department of Otolaryngology, National Cheng Kung University Hospital, College of Medicine, National Cheng Kung University, Tainan, Taiwan; 3 Department of Stomatology, National Cheng Kung University Hospital, College of Medicine, National Cheng Kung University, Tainan, Taiwan; 4 Department of Radiation Oncology, National Cheng Kung University Hospital, College of Medicine, National Cheng Kung University, Tainan, Taiwan; 5 Division of Hematology/Oncology, Department of Internal Medicine, National Cheng Kung University Hospital, College of Medicine, National Cheng Kung University, Tainan, Taiwan; 6 National Institute of Cancer Research, National Health Research Institutes, 1F No 367, Sheng-Li Road, Tainan, 70456 Taiwan

**Keywords:** Physical activity, Head and neck cancer, Case–control

## Abstract

**Background:**

Although substantial evidence supports a 20–30% risk reduction of colon cancer, breast cancer, and endometrial cancer by physical activity (PA), the evidence for head and neck cancer (HNC) is limited. Three published studies on the association between PA and HNC have generated inconsistent results. The current study examined the association between recreational PA (RPA) and HNC risk with a more detailed assessment on the intensity, frequency, duration, and total years of RPA.

**Methods:**

Data on RPA were collected from 623 HNC cases and 731 controls by in-person interview using a standardized questionnaire. The association between RPA and HNC risk was assessed using unconditional logistic regression, adjusted for sex, age, educational level, use of alcohol, betel quid, and cigarette, and consumption of vegetables and fruits.

**Results:**

A significant inverse association between RPA and HNC risk was observed in a logistic regression model that adjusted for sex, age, and education (odds ratio (OR) = 0.65, 95% confidence interval (CI): 0.51-0.82). However, after further adjustment for the use of alcohol, betel quid, and cigarette, and consumption of vegetables and fruits, RPA was no longer associated with HNC risk (OR =0.97, 95% CI: 0.73-1.28). No significant inverse association between RPA and HNC risk was observed in the analysis stratified by HNC sites or by the use of alcohol, betel quid, or cigarette.

**Conclusion:**

Results from our study did not support an inverse association between RPA and HNC risk. The major focus of HNC prevention should be on cessation of cigarette smoking and betel chewing, reduction of alcohol drinking, and promotion of healthy diet that contains plenty of fruits and vegetables.

**Electronic supplementary material:**

The online version of this article (doi:10.1186/s12885-017-3223-7) contains supplementary material, which is available to authorized users.

## Background

Head and neck cancer (HNC) (cancers of the oral cavity, oropharynx, hypopharynx, and larynx) is the fifth leading cancer in the world, with approximately 600,000 annual incident cases [1]. The majority of HNC cases are due to alcohol drinking, cigarette smoking, or betel quid chewing [2]. Recently, there is an increasing trend in the incidence of human papillomavirus-associated oropharyngeal cancer [3]. Studies of HNC have focused mostly on the risk factors and less information is available regarding factors associated with a decreased HNC risk. To date, only consumption of fruits and vegetables has been consistently associated with a reduced HNC risk [4].

Physical inactivity has been identified as the fourth leading contributor to global mortality [5]. The World Health Organization recommends adults 18–64 years old to perform at least 150 min of moderate-intensity aerobic physical activity (PA) or 75 min of vigorous-intensity aerobic PA per week [5]. Many studies have investigated the benefit of PA to reduce the risk of cancer. There is substantial evidence to support a 20–30% risk reduction of colon cancer, breast cancer, and endometrial cancer by PA, while the evidence for other cancers is limited [6, 7].

PA may have the potential to influence HNC risk by modulating the level of immunoglobulin A (IgA), which is the major class of antibodies in the fluids secreted by the mucosal surface, including saliva. IgA may serve as the first-line defense against foreign agents, including environmental carcinogens. It was shown that compared to the saliva of healthy controls, saliva of oral cancer patients had 45% lower level of IgA [8, 9].

To date, only three studies have investigated the association between PA and HNC risk and the results have been inconsistent. A cohort study by Leitzmann et al. reported a null association between recreational PA (RPA) and HNC risk while another cohort study by Hashibe et al. reported a significant inverse association between PA and HNC [10, 11]. A case–control study by Nicolotti et al. observed a 22% reduction in HNC risk with moderate RPA [12]. These studies did not have complete assessment of PA. Leitzmann et al. only examined the frequency (times per week) of PA [10]. Hashibe et al. only examined hours spent in vigorous activity per week [11], and Nicolotti et al. did not have sufficient information to calculate metabolic equivalent of task (MET) for evaluating dose–response relationship [12].

The current study examined the association between RPA and HNC risk with complete information on the intensity, frequency, duration, and total years of RPA.

## Methods

The institutional review boards of the National Health Research Institutes and the National Cheng Kung University Hospital approved this study. A signed informed consent was obtained from all participants of the study.

### Study subject recruitment

Data for the current analysis are from an ongoing HNC case–control study that began subject recruitment on September 1, 2010. Because questions on RPA were added later, the current analysis included subjects that were recruited from March 20, 2011 to October 29, 2015. Subject recruitment was conducted in the Department of Otolaryngology and the Department of Stomatology at the National Cheng Kung University Hospital. The eligibility criteria for the cases were: 1) pathologically confirmed diagnosis of squamous cell carcinoma of the head and neck, including cancers of the oral cavity, oropharynx, hypopharynx, and larynx; 2) no history of any type of cancer diagnosis; and 3) between the age of 20 and 80. Controls were recruited for comparing the risk of HNC and were selected by frequency-matching according to the sex and age (±5 years) distributions of the cases. The eligibility criteria for the controls were: 1) subjects who underwent surgery for non-cancerous conditions that are not associated with the consumption of alcohol, betel quid, and cigarette, with the most common diagnoses being benign lesions of the head and neck (oral cavity, oropharynx, hypopharynx, and larynx), chronic otitis media, chronic sinusitis, neck lipoma, obstructive sleep apnea, sialolithiasis, and thyroglossal duct cyst; 2) no history of any type of cancer diagnosis; and 3) between the age of 20 and 80.

### Data collection by interview

**Fig. 1:**
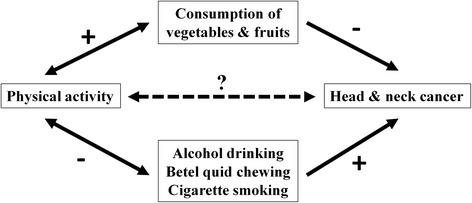
The proposed confounding structure for investigating the relationship between physical activity and head and neck cancer. “+” denotes a positive association, “-“ denotes an inverse association, and “?” denotes the association under investigation

Each study participant was interviewed by a trained interviewer using a standardized questionnaire to collect information on demographic characteristics (sex, age, and educational level) and regular RPA (Questions on RPA in Chinese can be seen on Additional file 1: Questionnaire). Each participant was asked whether he or she had been participating in RPA for at least three days a week, which we defined as regular RPA. Those with a positive response were further asked about the type of RPA, frequency (number of days per week), duration (number of hours per day), and the total years involved in each type of RPA. Individuals who engage in RPA may have a healthier lifestyle in general with less consumption of alcohol, betel quid, and cigarette and higher intake of vegetables and fruits, which have all been shown to influence HNC risk (Fig. 1). Therefore, to account for the potential confounding effect of other lifestyle factors, we also collected information on the use of alcohol, betel quid, and cigarette, and intake of vegetables and fruits. For alcohol, betel quid, and cigarette, detailed information was collected on starting age, quitting age (for former users), and dose (number of cigarettes per day, number of betel quids per day, and drinks of alcohol per week with each drink =150 ml of alcohol). For vegetables and fruits, participants were asked about the number of days per week that they ate vegetables or fruits.

### Statistical analysis

The distributions of demographic variables and lifestyle factors (alcohol drinking, betel quid chewing, cigarette smoking, and consumption of vegetables and fruits) between cases and controls were compared by performing T-tests (for continuous variables) and chi-squared tests (for categorical variables).

Odds ratio (OR) and 95% confidence interval (CI) were estimated to analyze the association between RPA and HNC risk using unconditional logistic regression, adjusted for sex, age, educational level, alcohol drinking (frequency), betel quid chewing (pack-years), cigarette smoking (pack-years), and consumption of vegetables and fruits (daily vs. non-daily). The pack-year of cigarette smoking = (number of cigarettes smoked per day/20) x number of years smoked. The pack-year of betel quid chewing = (number of betel quids chewed per day/20) x number of years chewed. We did not adjust for body mass index because we considered body mass index as an intermediate variable and not a confounder on the association between RPA and HNC risk. RPA was analyzed in several ways: 1) as a yes/no variable, with yes =3 or more days per week, no = less than 3 days per week; 2) by intensity: each type of RPA was assigned a MET value according to the 2011 Compendium of Physical Activities [13]. Each RPA was then assigned an intensity with light intensity =1.6-2.9 METs, moderate intensity =3.0-5.9 METs, and vigorous intensity =6.0 or more METs [14]. Individuals engaging in multiple RPAs with different levels of intensity were assigned the highest intensity among the multiple RPAs; 3) by frequency: no RPA (= less than 3 days per week), 3 days per week, 4–5 days per week, and 6–7 days per week; 4) by total MET-hours per week: for each individual, MET-hours per week was calculated for each type of RPA = MET for specific RPA x hours per day x days per week. Total MET-hours were then calculated by summing the MET-hours per week of all the RPAs performed for each individual; and 5) by the total of years RPA.

The development of HNC may influence an individual’s capability of performing RPA. To assess the possibility of reverse causality between RPA and HNC risk, sensitivity analysis was performed by censoring RPA at 5 years before the reference date (date of HNC diagnosis for the cases and date of interview for the controls).

Unconditional logistic regression was performed stratified by the use of alcohol, betel quid, or cigarette to examine the influence of alcohol, betel quid, or cigarette consumption on the association between RPA and HNC. Unconditional logistic regression model with the interaction term (RPA x alcohol, RPA x betel quid, or RPA x cigarette) was compared with the model without the interaction term by the log-likelihood ratio test to assess the heterogeneity between strata.

## Results

This analysis included 623 HNC cases and 731 controls. Cases and controls had similar mean age (55.4 years vs. 54.6 years, *P* = 0.20) (Table 1). Because the study is still ongoing with case–control frequency matching, case group had a higher percentage of women than the control group (6.7% vs. 2.5% *P* = 0.0001). More cases were users of alcohol, betel quid, and cigarette compared to controls (*P* < 0.0001). More controls ate vegetables and fruits daily than HNC cases (*P* < 0.0001).

**Table 1:** Demographic and lifestyle characteristics of the head and neck cancer patients and control subjects

Characteristics	Case *N* = 623 n (%)	Control *N* = 731 n (%)	*P*
Age (years)			
Mean (SE)	55.4 (0.4)	54.6 (0.4)	0.20
Sex			
Men	581 (93.3)	713 (97.5)	0.0001
Women	42 (6.7)	18 (2.5)	
Education			
≤ Elementary school	168 (27.0)	123 (16.8)	<0.0001
Junior high	185 (29.7)	133 (18.2)	
High school/Technical school	202 (32.4)	259 (35.4)	
Some college or more	68 (10.9)	216 (29.6)	
Alcohol drinking			
Never + occasional	196 (31.5)	385 (52.7)	<0.0001
Former regular	89 (14.3)	93 (12.7)	
Current regular	338 (54.2)	253 (34.6)	
Never	186 (29.9)	351 (48.0)	<0.0001
1 drink or less per month	10 (1.6)	34 (4.7)	
1-2 drinks per week	26 (4.2)	51 (7.0)	
3-5 drinks per week	32 (5.1)	44 (6.0)	
Daily drinkers	353 (56.6)	245 (33.5)	
Unknown	16 (2.6)	6 (0.8)	
Betel quid chewing			
Never	179 (28.7)	509 (69.7)	<0.0001
Former	235 (37.7)	141 (19.3)	
Current	209 (33.6)	80 (10.9)	
Unknown	0 (0.0)	1 (0.1)	
Never	179 (28.7)	509 (69.7)	<0.0001
0.1–9.9 pack-years	92 (14.8)	74 (10.1)	
10.0–19.9 pack-years	75 (12.0)	44 (6.0)	
20.0–29.9 pack-years	68 (10.9)	32 (4.4)	
30.0 or more pack-years	193 (31.0)	69 (9.4)	
Unknown	16 (2.6)	3 (0.4)	
Pack-years (SE)	26.8 (1.6)	8.3 (0.8)	<0.0001
Cigarette smoking			
Never	89 (14.3)	230 (31.5)	<0.0001
Former	117 (18.8)	146 (20.0)	
Current	416 (66.8)	354 (48.4)	
Unknown	1 (0.1)	1 (0.1)	
Never	89 (14.3)	230 (31.5)	<0.0001
0.1–9.9 pack-years	27 (4.3)	61 (8.3)	
10.0–19.9 pack-years	58 (9.3)	89 (12.2)	
20.0–29.9 pack-years	109 (17.5)	90 (12.3)	
30.0 or more pack-years	332 (53.3)	256 (35.0)	
Unknown	8 (1.3)	5 (0.7)	
Pack-years (SE)	35.8 (1.1)	23.8 (1.0)	<0.0001
Vegetable intake			
Non-daily	113 (18.1)	59 (8.1)	<0.0001
Daily	508 (81.6)	672 (91.9)	
Unknown	2 (0.3)	0 (0.0)	
Fruit intake			
Non-daily	434 (69.6)	334 (45.7)	<0.0001
Daily	186 (29.9)	396 (54.2)	
Unknown	3 (0.5)	1 (0.1)	

Abbreviations: *N* number, *SE* standard error

Among either HNC cases or controls, those who participated in regular RPA were less likely to consume alcohol, betel quid, or cigarette and more likely to eat vegetables and fruits daily (Table 2).

**Table 2:** The association between regular recreational physical activity and lifestyle characteristics by head and neck cancer status

	Case			Control		
Characteristics	No regular recreational physical activity *N* = 414 n (%)	Regular recreational physical activity *N* = 209 n (%)	*P*	No regular recreational physical activity *N* = 397 n (%)	Regular recreational physical activity *N* = 334 n (%)	*P*
Alcohol drinking						
Never + occasional	117 (28.3)	79 (37.8)	0.004	197 (49.6)	188 (56.3)	0.19
Former regular	53 (12.8)	36 (17.2)		55 (13.9)	38 (11.4)	
Current regular	244 (58.9)	94 (45.0)		145 (36.5)	108 (32.3)	
Never	111 (26.8)	75 (35.9)	0.07	177 (44.6)	174 (52.1)	0.02
1 drink or less per month	6 (1.4)	4 (1.9)		20 (5.0)	14 (4.2)	
1-2 drinks per week	17 (4.1)	9 (4.3)		29 (7.3)	22 (6.6)	
3-5 drinks per week	19 (4.6)	13 (6.2)		17 (4.3)	27 (8.1)	
Daily drinkers	252 (60.9)	101 (48.3)		151 (38.0)	94 (28.1)	
Unknown	9 (2.2)	7 (3.4)		3 (0.8)	3 (0.9)	
Betel quid chewing						
Never	93 (22.5)	86 (41.2)	<0.0001	246 (61.9)	263 (78.7)	<0.0001
Former	158 (38.1)	77 (36.8)		86 (21.7)	55 (16.5)	
Current	163 (39.4)	46 (22.0)		65 (16.4)	15 (4.5)	
Unknown	0 (0.0)	0 (0.0)		0 (0.0)	1 (0.3)	
Never	93 (22.5)	86 (41.2)	<0.0001	246 (61.9)	263 (78.7)	<0.0001
0.1–9.9 pack-years	59 (14.3)	33 (15.8)		49 (12.3)	25 (7.5)	
10.0–19.9 pack-years	49 (11.8)	26 (12.4)		28 (7.1)	16 (4.8)	
20.0–29.9 pack-years	54 (13.0)	14 (6.7)		21 (5.3)	11 (3.3)	
30.0 or more pack-years	147 (35.5)	46 (22.0)		51 (12.9)	18 (5.4)	
Unknown	12 (2.9)	4 (1.9)		2 (0.5)	1 (0.3)	
Pack-years (SE)	30.5 (2.1)	19.5 (2.3)	0.0004	10.8 (1.3)	5.3 (1.0)	0.0007
Cigarette smoking						
Never	43 (10.4)	46 (22.0)	<0.0001	101 (25.4)	129 (38.6)	<0.0001
Former	65 (15.7)	52 (24.9)		60 (15.1)	86 (25.8)	
Current	305 (73.7)	111 (53.1)		236 (59.5)	118 (35.3)	
Unknown	1 (0.2)	0 (0.0)		0 (0.0)	1 (0.3)	
Never	43 (10.4)	46 (22.0)	0.004	101 (25.4)	129 (38.6)	0.0004
0.1–9.9 pack-years	18 (4.4)	9 (4.3)		29 (7.3)	32 (9.6)	
10.0–19.9 pack-years	39 (9.4)	19 (9.1)		48 (12.1)	41 (12.3)	
20.0–29.9 pack-years	72 (17.4)	37 (17.7)		54 (13.6)	36 (10.8)	
30.0 or more pack-years	234 (56.5)	98 (46.9)		162 (40.8)	94 (28.1)	
Unknown	8 (1.9)	0 (0.0)		3 (0.8)	2 (0.6)	
Pack-years (SE)	37.8 (1.3)	32.0 (2.1)	0.02	27.5 (1.4)	19.4 (1.4)	<0.0001
Vegetable intake						
Non-daily	95 (22.9)	18 (8.6)	<0.0001	44 (11.1)	15 (4.5)	0.001
Daily	317 (76.6)	191 (91.4)		353 (88.9)	319 (95.5)	
Unknown	2 (0.5)	0 (0.0)		0 (0.0)	0 (0.0)	
Fruit intake						
Non-daily	328 (79.2)	106 (50.7)	<0.0001	229 (57.7)	105 (31.4)	<0.0001
Daily	83 (20.1)	103 (49.3)		167 (42.1)	229 (68.6)	
Unknown	3 (0.7)	0 (0.0)		1 (0.2)	0 (0.0)	

In the unconditional logistic regression model adjusted for sex, age, and education (Model 1), RPA was associated with a significantly decreased HNC risk (OR =0.65, 95% CI = 0.51-0.82) (Table 3). After additional adjustment for consumption of alcohol, betel quid, and cigarette (Model 2) the OR moved toward the null and became non-statistically significant (OR =0.83, 95%: 0.64-1.09). Further adjustment for daily intake of vegetables and fruits (Model 3) generated a null association between RPA and HNC risk (OR =0.97, 95% CI: 0.73-1.28). For the intensity of RPA, the model with adjustment for sex, age, and education showed an inverse trend between the intensity of RPA and HNC risk with moderate and vigorous intensity being associated with a significantly reduced HNC risk (moderate intensity: OR =0.72, 95% CI: 0.53-0.98; vigorous intensity: OR =0.57, 95% CI: 0.42-0.77). However, after additional adjustment for alcohol, betel quid, cigarette, vegetables, and fruits, the reduced HNC risk associated with moderate intensity RPA became null (OR =1.09, 95% CI: 0.77-1.54) and the reduced HNC risk associated with vigorous intensity RPA became non-statistically significant (OR =0.85, 95% CI: 0.60-1.22). The analyses with RPA frequency, total MET-hours per week, and total years all showed a significant inverse association with HNC risk in models adjusted for sex, age, and education, although a dose–response relationship was not apparent. After further adjustment for alcohol, betel quid, cigarette, vegetables, and fruits, no significant association was found between HNC risk and RPA frequency, total MET-hours per week, or total years.

**Table 3:** The association between regular recreational physical activity and head and neck cancer

Regular recreational physical activity	Case *N* = 623 n (%)	Control *N* = 731 n (%)	Model 1^a^ OR (95% CI)	Model 2^b^ OR (95% CI)	Model 3^c^ OR (95% CI)
Yes/No					
No regular exercise	414 (66.5)	397 (54.3)	Reference	Reference	Reference
Regular exercise	209 (33.5)	334 (45.7)	0.65 (0.51–0.82)	0.83 (0.64–1.09)	0.97 (0.73–1.28)
Intensity					
No regular exercise	414 (66.5)	397 (54.3)	Reference	Reference	Reference
light	10 (1.6)	10 (1.4)	0.88 (0.34–2.25)	0.98 (0.36–2.65)	1.07 (0.39–2.92)
moderate	114 (18.3)	154 (21.1)	0.72 (0.53–0.98)	0.95 (0.68–1.33)	1.09 (0.77–1.54)
vigorous	85 (13.6)	170 (23.3)	0.57 (0.42–0.77)	0.72 (0.51–1.02)	0.85 (0.60–1.22)
Frequency					
No regular exercise	414 (66.5)	397 (54.3)	Reference	Reference	Reference
3 days per week	38 (6.1)	60 (8.2)	0.76 (0.49-1.19)	1.13 (0.69-1.85)	1.29 (0.78-2.14)
4–5 days per week	26 (4.2)	54 (7.4)	0.50 (0.30-0.83)	0.72 (0.41-1.27)	0.83 (0.47-1.47)
6–7 days per week	145 (23.2)	220 (30.1)	0.66 (0.50-0.86)	0.79 (0.58-1.07)	0.93 (0.68-1.27)
Total MET-hours per week					
No regular exercise	414 (66.5)	397 (54.3)	Reference	Reference	Reference
0.1–10.0	43 (6.9)	64 (8.8)	0.63 (0.41–0.98)	0.84 (0.52–1.37)	0.95 (0.58–1.55)
10.1–20.0	63 (10.1)	91 (12.4)	0.68 (0.47–0.98)	0.82 (0.55–1.24)	0.93 (0.61–1.41)
20.1–30.0	38 (6.1)	64 (8.8)	0.63 (0.40–0.98)	0.82 (0.50–1.34)	0.97 (0.59–1.59)
> 30.0	64 (10.3)	115 (15.7)	0.64 (0.45–0.91)	0.84 (0.56–1.24)	1.02 (0.68–1.54)
Unknown	1 (0.1)	0 (0.0)	--	--	--
Total years of regular exercise					
No regular exercise	414 (66.5)	397 (54.3)	Reference	Reference	Reference
0.1–5.0	114 (18.3)	165 (22.6)	0.71 (0.53–0.94)	0.80 (0.58–1.10)	0.90 (0.64–1.25)
5.1–10.0	46 (7.4)	92 (12.6)	0.51 (0.34–0.76)	0.66 (0.42–1.03)	0.82 (0.52–1.28)
> 10	49 (7.8)	77 (10.5)	0.69 (0.46–1.05)	1.28 (0.80–2.03)	1.54 (0.96–2.49)

Abbreviations: *CI* confidence interval, *N* number, *OR* odds ratio

^a^Model 1: OR and 95% CI were calculated using unconditional logistic regression, adjusted for sex, age, and education

^b^Model 2: OR and 95% CI were calculated using unconditional logistic regression, adjusted for sex, age, education, cigarette smoking (pack-year categories), betel quid chewing (pack-year categories), and alcohol drinking (frequency)

^c^Model 3: OR and 95% CI were calculated using unconditional logistic regression, adjusted for sex, age, education, cigarette smoking (pack-year categories), betel quid chewing (pack-year categories), alcohol drinking (frequency), and intake of vegetables and fruits

We performed sensitivity analysis by censoring RPA at 5 years before the reference date (date of HNC diagnosis for the cases and date of interview for the controls). The result showed a null association between RPA censored at 5 years before the reference date and HNC risk (OR =1.08, 95% CI = 0.77-1.51).

No significant association was observed between RPA (yes/no, intensity, frequency, and total MET-hours per week) and HNC risk in analyses stratified by HNC sites (Table 4). No significant association between total years of RPA and risk of pharyngeal cancer or laryngeal cancer was observed. A positive association was found between >10 years of RPA and oral cancer risk (OR =1.87, 95% CI: 1.06-3.28).

**Table 4:** The association between regular recreational physical activity and head and neck cancer by disease site

		Oral Cancer		Pharyngeal Cancer		Laryngeal Cancer	
Regular recreational physical activity	Control *N* = 731 n (%)	Cases *N* = 395 n (%)	OR (95% CI)^a^	Cases *N* = 154 n (%)	OR (95% CI)^a^	Cases *N* = 74 n (%)	OR (95% CI)^a^
Yes/No							
No regular exercise	397 (54.3)	265 (67.1)	Reference	107 (69.5)	Reference	42 (56.8)	Reference
Regular exercise	334 (45.7)	130 (32.9)	1.02 (0.74–1.41)	47 (30.5)	0.79 (0.49–1.27)	32 (43.2)	1.03 (0.58–1.85)
Intensity							
No regular exercise	397 (54.3)	265 (67.1)	Reference	107 (69.5)	Reference	42 (56.8)	Reference
light	10 (1.4)	5 (1.3)	0.91 (0.26–3.12)	1 (0.6)	0.27 (0.3–2.49)	4 (5.4)	2.45 (0.61–9.95)
moderate	154 (21.1)	71 (17.9)	1.19 (0.80–1.78)	26 (16.9)	0.89 (0.49–1.61)	17 (23.0)	0.93 (0.46–1.87)
vigorous	170 (23.3)	54 (13.7)	0.87 (0.58–1.32)	20 (13.0)	0.75 (0.41–1.38)	11 (14.8)	1.01 (0.45–2.24)
Frequency							
No regular exercise	397 (54.3)	265 (67.1)	Reference	107 (69.5)	Reference	42 (56.8)	Reference
3 days per week	60 (8.2)	23 (5.8)	1.34 (0.73–2.44)	11 (7.1)	1.44 (0.65–3.19)	4 (5.4)	1.19 (0.36–3.96)
4–5 days per week	54 (7.4)	21 (5.3)	1.02 (0.54–1.92)	4 (2.6)	0.51 (0.16–1.59)	1 (1.3)	0.32 (0.04–2.55)
6–7 days per week	220 (30.1)	86 (21.8)	0.95 (0.66–1.38)	32 (20.8)	0.70 (0.41–1.22)	27 (36.5)	1.14 (0.61–2.12)
Total MET-hours per week							
No regular exercise	397 (54.3)	265 (67.1)	Reference	107 (69.5)	Reference	42 (56.8)	Reference
0.1-10.0	64 (8.8)	29 (7.3)	1.07 (0.61–1.90)	11 (7.1)	0.90 (0.40–2.01)	3 (4.0)	0.43 (0.12–1.60)
10.1-20.0	91 (12.4)	37 (9.4)	0.94 (0.58–1.53)	13 (8.4)	0.59 (0.28–1.23)	13 (17.6)	1.14 (0.52–2.51)
20.1-30.0	64 (8.8)	22 (5.6)	0.91 (0.50–1.65)	8 (5.2)	1.03 (0.43–2.48)	8 (10.8)	1.37 (0.54–3.47)
> 30.0	115 (15.7)	42 (10.6)	1.16 (0.72–1.87)	15 (9.8)	0.84 (0.42–1.70)	7 (9.5)	1.10 (0.42–2.87)
Unknown	0 (0.0)	0 (0.0)	--	0 (0.0)	--	1 (1.3)	--
Total years of regular exercise							
No regular exercise	397 (54.3)	265 (67.1)	Reference	107 (69.5)	Reference	42 (56.8)	Reference
0.1-5.0	165 (22.6)	76 (19.2)	0.97 (0.67–1.42)	24 (15.6)	0.71 (0.41–1.25)	14 (18.9)	0.79 (0.39–1.62)
5.1-10.0	92 (12.6)	24 (6.1)	0.70 (0.40–1.24)	12 (7.8)	0.92 (0.43–1.96)	10 (13.5)	1.74 (0.73–4.14)
> 10.0	77 (10.5)	30 (7.6)	1.87 (1.06–3.28)	11 (7.1)	0.91 (0.39–2.11)	8 (10.8)	1.14 (0.41–3.14)

Abbreviations: *CI* confidence interval, *N* number, *OR* odds ratio

^a^OR and 95% CI were calculated using unconditional logistic regression, adjusted for sex, age, education, cigarette smoking (pack-year categories), betel quid chewing (pack-year categories), alcohol drinking (frequency), and intake of vegetables and fruits

In analysis stratified by the use of alcohol, betel quid, or cigarette, no significant association was found between RPA and HNC risk (Table 5).

**Table 5:** The association between regular recreational physical activity and risk of head and neck cancer stratified by the use of alcohol, betel quid, or cigarette

	No regular recreational physical activity vs. regular recreational physical activity OR (95% CI)^a^
Alcohol drinking	
Never + occasional	0.97 (0.63–1.49)
Former regular	1.34 (0.59–3.04)
Current regular	0.99 (0.64–1.53)
Former regular + current regular	1.05 (0.72–1.52)
	P-interaction =0.83
Betel quid	
Never	0.95 (0.63–1.43)
Former	0.79 (0.49–1.28)
Current	1.96 (0.91–4.21)
Former + Current	1.04 (0.70–1.53)
	P-interaction =0.75
Cigarette	
Never	1.59 (0.81–3.10)
Former	0.73 (0.40–1.33)
Current	0.96 (0.66–1.40)
Former + Current	0.92 (0.67–1.25)
	P-interaction =0.61

Abbreviations: *CI* confidence interval, *OR* odds ratio

^a^OR and 95% CI were calculated using unconditional logistic regression, adjusted for sex, age, education, cigarette smoking (pack-year categories), betel quid chewing (pack-year categories), alcohol drinking (frequency), and intake of vegetables and fruits

## Discussion

In the current analysis, we found a significant inverse association between RPA and HNC risk in the logistic regression model that adjusted for sex, age, and education. However, after further adjustment for the use of alcohol, betel quid, and cigarette, and consumption of vegetables and fruits, RPA was no longer associated with HNC risk. No significant inverse association between RPA and HNC risk was observed in the analysis stratified by HNC sites or by the use of alcohol, betel quid, or cigarette.

To date, three studies have been published on the association between PA and HNC and the results have been inconsistent. Leitzmann et al. examined the association between RPA and HNC risk in a cohort of 487,732 subjects [10]. They found that individuals who engaged in RPA five or more times per week had a reduced HNC risk (relative risk (RR) = 0.62, 95% CI: 0.52-0.74) compared to those who performed RPA less than once per month in a statistical model that adjusted for age and sex only [10]. After including smoking as an additional covariate, the RR moved substantially toward the null and became non-statistically significant (RR = 0.86, 95% CI: 0.72-1.03) [10]. Further adjustment for body mass index, race/ethnicity, marital status, family history of any cancer, education, intake of fruits and vegetables, red meat, and alcohol only had a small impact (RR = 0.89, 95% CI = 0.74-1.06) [10]. In another cohort study, Hashibe et al. evaluated the development of HNC by PA status in a cohort of 101,182 subjects [11]. With PA information available for less than half of the subjects, they observed a significantly reduced HNC risk for those who participated in 3 or more hours of vigorous activity at baseline interview compared to those who had <1 h of vigorous activity at baseline interview (RR = 0.58, 95% CI: 0.35-0.96), adjusted for age, sex, race, education, drinking frequency, and tobacco pack-years [11]. When PA was examined at age 40, those who participated in 3 or more hours of vigorous activity at age 40 had a non-significantly reduced HNC risk compared to those who had <1 h of vigorous activity at age 40 (RR = 0.69, 95% CI: 0.42-1.14), adjusted for age, sex, race, education, drinking frequency, and tobacco pack-years [11]. In a pooled case–control study of 2289 HNC cases and 5580 controls, Nicolotti et al. reported that moderate RPA was associated with a significantly reduced HNC risk (OR =0.78, 95%: 0.66-0.91) and high RPA was associated with a non-significantly reduced HNC risk (OR =0.72, 95% CI: 0.46-1.16), adjusted for age, sex, study center, ethnicity, education, occupational PA, cigarette smoking and alcohol drinking [12].

In the investigation for the association between PA and HNC, it would be important to adjust for other lifestyle factors that have been strongly associated with an increased HNC risk, including use of alcohol, betel quid, and cigarette, and reduced consumption of fruits and vegetables [2, 4]. Individuals who participate in PA tend to have different health behavior patterns from individuals who live a sedentary lifestyle [15, 16]. In our analysis, we found that individuals who engaged in RPA were less likely to drink alcohol, chew betel quid, and smoke cigarette and more likely to eat fruits and vegetables everyday. When we adjusted for sex, age, and education only, we observed a significant inverse association between RPA and HNC risk. However, this inverse association became null after we further adjusted for use of alcohol, betel quid, and cigarette, and consumption of vegetables and fruits. This indicated that the inverse association between RPA and HNC was cofounded by these other lifestyle factors and RPA was not independently associated with HNC. The two studies that found a significant inverse association between PA and HNC did not adjust for intake of fruits and vegetables and there could be residual confounding for the association in these studies [11, 12].

When we examined the association between RPA and HNC risk by HNC sites, we didn’t find any significant association except for the positive association between >10 years of RPA and oral cancer risk. It is unclear why higher total years of RPA would be associated an increased oral cancer risk. Because of the smaller numbers in the stratified analysis, chance finding could not be ruled out. Leitzmann et al. did not find a significant association between RPA (5 more times of RPA per week vs. no physical activity) and any of the HNC sites (Oral cavity: RR = 0.98, 95% CI: 0.75-1.29; pharynx: RR = 0.70, 95% CI: 0.45-1.08; larynx: RR = 0.82, 95% CI: 0.59-1.13) [10]. Nicolotti et al. reported an inverse association between moderate RPA and oral cancer (OR =0.74, 95% CI: 0.56-0.97) and pharyngeal cancer (OR =0.67, 95% CI: 0.53-0.85) [12]. In addition, they found that high RPA was associated with a reduced risk of oral cancer risk (OR =0.53, 95% CI: 0.32-0.88) and pharyngeal cancer (OR =0.58, 95% CI: 0.38-0.89) but an increased risk of laryngeal cancer (OR =1.73, 95% CI: 1.04-2.88) [12]. Again, the reduced risk reported by Nicolotti could be attributed partly to the residual confounding by not adjusting for intake of fruits and vegetables. According to Nicolotti et al., the increased laryngeal cancer risk associated with high RPA levels could be due to residual confounding by cigarette smoking because of the higher percentage of cigarette smokers among laryngeal cancer patients with high PA levels [12].

We examined whether the association between RPA and HNC risk could be modified by the use of alcohol, betel quid, or cigarette. Our results did not indicate any effect modification of these lifestyle factors on the association between RPA and HNC. Leitzmann et al. showed the inverse association between RPA and HNC risk was more evident among ever alcohol drinkers than among never alcohol drinkers (P for heterogeneity between strata =0.03) [10]. Nicolotti showed that the reduced HNC risk associated with moderate RPA was more evident among ever tobacco smokers and ever alcohol drinkers, although it was not statistically significant between the strata (P for heterogeneity between strata =0.25) [12]. Given the inconsistencies among studies, further investigations are needed to determine whether RPA is beneficial for certain subgroups, in particular alcohol drinkers and cigarette smokers, for reducing HNC risk.

This study has several limitations. Because case–control studies collect exposure data by asking participants to recall their past exposures or activities, there can be recall bias and recall error. Recall bias often occurs when the case subjects ruminate on the exposure that may possibly cause their development of disease, resulting in a spurious positive association between exposure and the disease. However, this may not be a major issue for our study because we found a null association between RPA and HNC risk. Since the public is not aware of the possible association between RPA and HNC, non-differential random recall error was more likely for our study and could have biased our results toward the null. Another limitation is that we did not collect information on occupation and thus could not adjust for occupational PA in our statistical models. Finally, although human papillomavirus is an important risk factor for oropharyngeal cancer, we did not have access to the tumor tissue to test for HPV status. For HNC occurring in the oral cavity, hypopharynx, and larynx, the contribution of HPV is likely very low [17]. We conducted an additional sensitivity analysis focusing on two HNC sites (tonsil and base of the tongue) that show the strongest association with HPV [18]. We did not see an association between RPA and cancers of the tonsil and the base of the tongue (Additional file 2: Table S1). In addition, no population-based study has been conducted in Taiwan to assess the contribution of HPV to the development of oropharyngeal cancer. A study from Taiwan with 111 samples of tonsillar squamous cell carcinoma found that only 12.6% of the samples were HPV positive [19]. Overall, we think that HPV status made minimal impact on our results showing a null association between RPA and HNC.

The major strength of the current study is the detailed assessment of RPA. We collected information on the type, intensity, frequency, and duration of RPA. This allowed us to be the first study to calculate MET-hours for evaluating the dose–response relationship between RPA and HNC risk. Another strength is that we adjusted for lifestyle factors that have been strongly associated with HNC risk, including use of alcohol, betel quid, and cigarette, and consumption of vegetables and fruits. This minimized the possibility of confounding on the association between RPA and HNC risk by other health behaviors.

## Conclusions

In conclusion, results from our study did not support an inverse association between RPA and HNC risk. Although RPA is beneficial in reducing the risk of various chronic diseases and certain cancers, including colon cancer, breast cancer, and endometrial cancer [6, 7], our results suggested that RPA is unlikely to play a major role to reduce HNC risk. The major focus of HNC prevention should be on cessation of cigarette smoking and betel chewing, reduction of alcohol drinking, and promotion of healthy diet that contains plenty of fruits and vegetables.

## Additional files

Additional file 1: Questionnaire. Physical activity questions. This file contains questions used to collect physical activity data (DOC 29 kb)

Additional file 2: Table S1. The association between regular recreational physical activity and cancers of tonsil and tongue base and other pharyngeal cancers. This supplementary table examines the association between regular recreational physical activity and head and neck cancer sites by the association with human papillomavirus. (DOC 75 kb)

## Abbreviations

CI: Confidence interval
HNC: Head and neck cancer
HPV: Human papillomavirus
OR: Odds ratio
PA: Physical activity
RPA: Recreational physical activity
