# Supplementary material for: Regular recreational physical activity and risk of head and neck cancer
Source: BMC Cancer. 2017 Apr 21;17:286. doi: 10.1186/s12885-017-3223-7 (PMC5399847; doi:10.1186/s12885-017-3223-7)
Supplement: Supplementary file 1 — Questionnaire. Physical activity questions. This file contains questions used to collect physical activity data (DOC 29 kb) [file 12885_2017_3223_MOESM1_ESM.doc]

**Questionnaire S1. Physical activity questions**

**運動習慣之調查**

**請問您有運動的習慣嗎？(一周至少運動三天以上)**

**□(01)有**

**□(02)沒有**

**□(98)不知道**

□(99)拒絕回答

| **運動名稱** | **天/一周** | **小時/天** | **持續多久 (年)** |
| --- | --- | --- | --- |
|  |  |  |  |
|  |  |  |  |
|  |  |  |  |

**請問您最常做的三種運動名稱及頻率為何？**
