# Supplementary material for: Regular recreational physical activity and risk of head and neck cancer
Source: BMC Cancer. 2017 Apr 21;17:286. doi: 10.1186/s12885-017-3223-7 (PMC5399847; doi:10.1186/s12885-017-3223-7)
Supplement: Supplementary file 2 — The association between regular recreational physical activity and cancers of tonsil and tongue base and other pharyngeal cancers. This supplementary table examines the association between regular recreational physical activity and head and neck cancer sites by the association with human papillomavirus. (DOC 75 kb) [file 12885_2017_3223_MOESM2_ESM.doc]

Table S1. The association between regular recreational physical activity and cancers of tonsil and tongue base and other pharyngeal cancers

|  |  | **Cancers of tonsil and tongue Base** | | **Other pharyngeal cancers** | |
| --- | --- | --- | --- | --- | --- |
| **Regular recreational physical activity** | **Control**  **N=731**  **n (%)** | **Cases**  **N=46**  **n (%)** | **OR (95% CI)^a^** | **Cases**  **N=108**  **n (%)** | **OR (95% CI)^a^** |
| **Yes/No** |  |  |  |  |  |
| No regular exercise | 397 (54.3) | 29 (63.0) | Referent | 78 (72.2) | Referent |
| Regular exercise | 334 (45.7) | 17 (37.0) | 0.81 (0.39-1.70) | 30 (27.8) | 0.81 (0.46-1.43) |
|  |  |  |  |  |  |
| **Intensity** |  |  |  |  |  |
| No regular exercise | 397 (54.3) | 29 (63.0) | Referent | 78 (72.2) | Referent |
| light | 10 (1.4) | 0 (0.0) | -- | 1 (0.9) | 0.66 (0.07-6.48) |
| moderate | 154 (21.1) | 6 (13.0) | 0.48 (0.17-1.37) | 20 (18.5) | 1.17 (0.58-2.33) |
| vigorous | 170 (23.3) | 11 (23.9) | 1.39 (0.59-3.27) | 9 (8.3) | 0.52 (0.23-1.18) |
|  |  |  |  |  |  |
| **Frequency** |  |  |  |  |  |
| No regular exercise | 397 (54.3) | 29 (63.0) | Referent | 78 (72.2) | Referent |
| 3 days per week | 60 (8.2) | 4 (8.7) | 1.54 (0.45-5.27) | 7 (6.5) | 1.60 (0.61-4.25) |
| 4-5 days per week | 54 (7.4) | 2 (4.4) | 0.66 (0.13-3.31) | 2 (1.9) | 0.36 (0.07-1.77) |
| 6-7 days per week | 220 (30.1) | 11 (23.9) | 0.69 (0.29-1.64) | 21 (19.4) | 0.75 (0.39-1.44) |
|  |  |  |  |  |  |
| **Total MET-hours per week** |  |  |  |  |  |
| No regular exercise | 397 (54.3) | 29 (63.0) | Referent | 78 (72.2) | Referent |
| 0.1-10.0 | 64 (8.8) | 2 (4.4) | 0.33 (0.06-1.71) | 9 (8.3) | 1.45 (0.57-3.67) |
| 10.1-20.0 | 91 (12.4) | 6 (13.0) | 0.84 (0.30-2.33) | 7 (6.5) | 0.48 (0.18-1.24) |
| 20.1-30.0 | 64 (8.8) | 3 (6.5) | 1.36 (0.35-5.25) | 5 (4.6) | 1.00 (0.34-2.94) |
| > 30.0 | 115 (15.7) | 6 (13.0) | 1.01 (0.34-3.05) | 9 (8.3) | 0.78 (0.33-1.87) |
| Unknown | 0 (0.0) | 0 (0.0) | -- | 0 (0.0) | -- |
|  |  |  |  |  |  |
| **Total years of regular exercise** |  |  |  |  |  |
| No regular exercise | 397 (54.3) | 29 (63.0) | Referent | 78 (72.2) | Referent |
| 0.1-5.0 | 165 (22.6) | 8 (17.4) | 0.74 (0.30-1.80) | 16 (14.8) | 0.73 (0.36-1.44) |
| 5.1-10.0 | 92 (12.6) | 3 (6.5) | 0.53 (0.13-2.16) | 9 (8.3) | 1.18 (0.48-2.90) |
| > 10.0 | 77 (10.5) | 6 (13.0) | 1.58 (0.50-5.05) | 5 (4.6) | 0.67 (0.22-2.06) |

Abbreviations: CI: confidence interval, N: number, OR: odds ratio

1. OR and 95% CI were calculated using unconditional logistic regression, adjusted for sex, age, education, cigarette smoking (pack-year categories), betel quid chewing (pack-year categories), alcohol drinking (frequency), and intake of vegetables and fruits
